# Intra-operative cerebrospinal fluid sampling versus post-operative lumbar puncture for detection of leptomeningeal disease in malignant paediatric brain tumours

**DOI:** 10.1371/journal.pone.0196696

**Published:** 2018-05-03

**Authors:** Sharon Y. Y. Low, Chen Min Wei, Kenneth T. E. Chang, Chan Yiong Huak, Ng Lee Ping, Seow Wan Tew, David C. Y. Low

**Affiliations:** 1 Neurosurgical Service, KK Women’s and Children’s Hospital, Singapore, Singapore, Singapore; 2 Department of Neurosurgery, National Neuroscience Institute, Singapore, Singapore, Singapore; 3 SingHealth Duke-NUS Neuroscience Academic Clinical Program, Singapore, Singapore; 4 Dept of Pathology & Laboratory Medicine, KK Women’s and Children’s Hospital, Singapore, Singapore, Singapore; 5 Biostatistics Unit, Yong Loo Lin School of Medicine, National University of Singapore, National University Health System, Singapore, Singapore; BIDMC, UNITED STATES

## Abstract

**Introduction:**

Leptomeningeal disease is a feared sequelae of malignant paediatric brain tumours. Current methods for its detection is the combined use of cranio-spinal MRI, and CSF cytology from a post-operative lumbar puncture. In this study, the authors hypothesize that CSF taken at the start of surgery, either from an external ventricular drain or neuroendoscope will have equal sensitivity for positive tumour cells, in comparison to lumbar puncture. Secondary hypotheses include positive correlation between CSF cytology and MRI findings of LMD. From a clinical perspective, the key aim of the study was for affected paediatric patients to avoid an additional procedure of a lumbar puncture, often performed under anaesthesia after neurosurgical intervention.

**Methods:**

This is single-institution, retrospective study of paediatric patients diagnosed with malignant brain tumours. Its main aim was to compare cytological data from CSF collected at the time of surgery versus data from an interval lumbar puncture. In addition, MRI imaging of the same cohort of patients was examined for leptomeningeal disease and corroborated against CSF tumour cytology findings.

**Results:**

Thirty patients are recruited for this study. Data analysis demonstrates a statistically significant association between our intra-operative CSF and LP sampling. Furthermore, our results also show for significant correlation between evidence of leptomeningeal disease on MRI findings versus intra-operative CSF positivity for tumour cells.

**Conclusion:**

Although this is a retrospective study with a limited population, our data concurs with potential to avoid an additional procedure for the paediatric patient diagnosed with a malignant brain tumour.

## Introduction

Leptomeningeal disease (LMD) is a feared sequelae of malignant paediatric brain tumours. Active perquisition of their existence is an important priority in affected patients. At present, the recommended method for LMD detection is the combinatorial use of gadolinium-enhanced magnetic resonance imaging (MRI) of the neuroaxis, and the cytologic examination of cerebrospinal fluid (CSF) specimens obtained within 2 to 3 weeks after surgery. This practice, utilised by our institution, is largely extrapolated from Gajjar *et al* [1]. As part of our workflow, affected patients usually undergo a post-operative lumbar puncture (LP). If the child is too young, or has challenging spinal anatomy, the LP may be performed under image-guidance. In contrast to adult patients, most of these procedures need to be performed under sedation or even anaesthesia as children can be restless and thus, uncooperative during LP. Recently, published studies have raised concerns that anaesthetics may cause neurotoxic changes in the developing brain, leading to adverse neurodevelopmental outcomes [2].

Paediatric brain tumour patients commonly present with symptoms secondary to obstructive hydrocephalus, owing to the frequent midline, peri-circumventricular locations of their lesions. Here, hydrocephalus results from direct mechanical blockade of CSF flow from the primary tumour, often corroboratively with LMD, if the latter is present [3]. Under such circumstances, it is usual practice for the operating neurosurgeon to either insert an external ventricular drain (EVD) at the start of surgery; or decant excess CSF via a neuro-endoscope placed into the engorged ventricle. Such manoeuvres aim to firstly, reduce intracranial pressure and next, achieve adequate brain relaxation for surgery to proceed. At present, there is no study to compare the positivity of CSF cytology taken *at the beginning of surgery*, versus interval samples taken 2 to 3 weeks post-operatively via lumbar puncture. Given that CSF cytology is hypothesized to be dependent on neoplastic cells being shed from the primary tumour source, a logical assumption will be that at this time-point, a high pickup rate will be more likely—tumour at its largest and most number of cells. Given that false-negative detection of malignant cells on cytology is a common occurrence, higher CSF volumes will theoretically increase positive yield. Some studies have quoted a CSF volume of at least 10.5 ml to have feasible detection of malignant cells [4, 5]. Unfortunately, high volumes via LP in a young child may be unrealistic. In contrast, intra-operative CSF decanted to achieve brain relaxation will be more likely to produce higher volumes for detection.

For this study, the authors hypothesize that CSF collected during at the start of brain tumour surgery will be reflective of overall LMD in affected patients. The primary objective for this project is to demonstrate that the sensitivity of CSF cytology when sampled intracranially at the beginning will be corroborative with post-operative CSF findings from lumbar puncture. Secondary objectives include the assessment of correlation between pre-operative MRI results with CSF results for LMD in the same cohort of patients.

## Methods

### Study design and patient demographics

This is an ethics-approved, single-institution, retrospective study of patients aged less than 18 years old under the care of the Neurosurgical Service, KKH. The study is designed for proof-of-concept: focusing on the comparison of cytological data from CSF collected at the time of surgery versus data from an interval LP. Inclusion criteria involved all paediatric patients with malignant brain tumours who had a ventricular drain, or a neuro-endoscope inserted at the time of surgery, prior to tumour resection or biopsy. Following that, the same patient would have undergone LP up to 60 days post-operatively. Cerebrospinal fluid collected at both events were sent for cytology. For the purposes of this manuscript, ‘malignant brain tumours’ is defined as WHO III and IV as per WHO 2016 classification [6]. These tumours were selected owing to their reputed risks of metastasizing along the neuro-axis [7–11]. Exclusion criteria included patients with WHO I or II brain tumours [6], whose risk of CSF seeding or metastasis was low. Other excluded candidates for this study were those whose cranial CSF was collected from permanent implants such as a ventriculo-peritoneal shunt or Ommaya reservoir, patients who underwent LP procedures performed more than 30 days post-operatively, and patients with incomplete data. The ethics committee waived patient consent requirement for this study. All selected patients were anonymised using coded de-identifiers for data protection. Only the approved study team members had access to the participants’ information for analysis.

### MRI cranio-spinal axis

**Fig 1:**
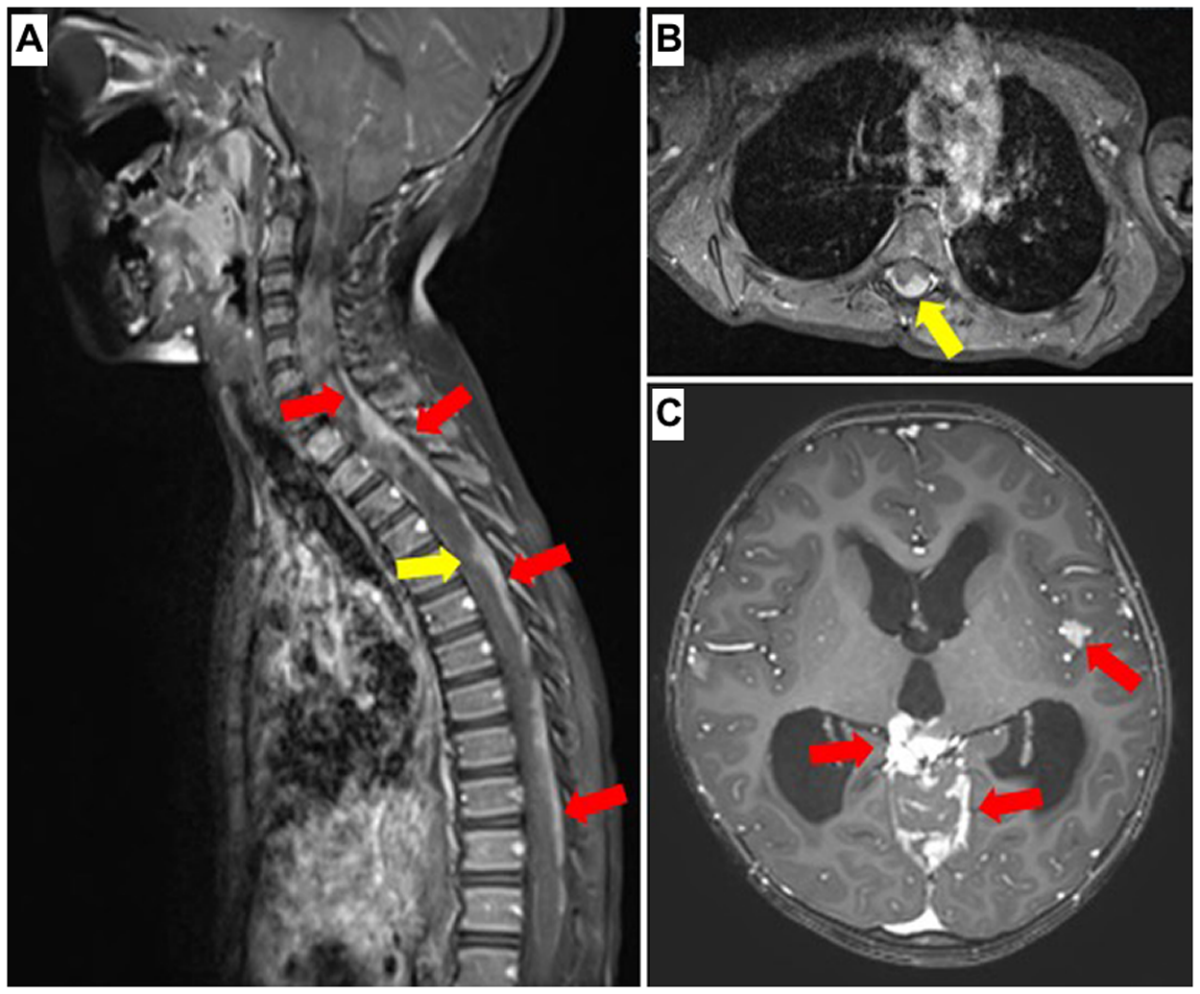
(**A**) Post-contrast T1-weighted MRI cervico-thoracic spine demonstrating extensive leptomeningeal enhancement along vertical axis of spinal dura. Red and yellow arrows depict leptomeningeal disease consistent with metastasis. (B) Yellow arrow correlates to same level of disease in the post-contrast T1-weighted axial image. (**C**) Representative image of T1-weighted post-contrast axial MRI with leptomeningeal enhancement, contrast-enhancement over areas in a‘sugar-coated’ manner and nodular disease, as highlighted by the red arrows.

All patients underwent a pre-operative MRI of their whole neuroaxis, with T1-post-gadonilium imaging as part of the sequences. The MRI sequences for the brain and spine were performed on either 1.5 Tesla scanner (GE^™^ Signa on HD23 platform) or 3 Tesla scanner (Siemens^™^ Skyra on VE 11A platform) at our institution. Post-contrast T1-weighted images depicting leptomeningeal enhancement, (that is, diffuse or focal gyriform or serpentine enhancement on the dural and, or leptomeninges) was the primary mode of diagnosis. Findings of contrast-enhancement over cranio-spinal areas in a‘sugar-coated’ manner are also included as positive findings. In addition, the MRI images were screened for presence of lesions along the cranio-spinal sites away from the primary tumour of interest. (Fig 1)

### Intra-operative CSF collection

Under general anaesthesia, the patient was positioned as required by the operating neurosurgeon for surgery. To achieve brain relaxation, an EVD was inserted at either Kocher’s (coronal) point, or Frazier’s (parieto-occipital) point [12]. For neuroendoscopy procedures, usually a pre-coronal burr hole was made in the mid-pupillary line. Next, a rigid zero-degree, neuroendoscope was introduced transcortically into the ventricle to visualise the tumour. At the point of *first* ventricular cannulation, CSF was immediately collected for cytology. Depending on the patient’s age, aetiology of the tumour and extent of hydrocephalus, an estimated 10 to 20 mL of CSF is usually obtained. The key point to note here, would be the *timing* of the cannulation into a previously untouched ventricle, and not at an interval point later in the surgery. This was because sterile wash would be often used intra-operatively as part of the procedure, and could potentially dilute CSF to cause an inaccurate result.

### Lumbar puncture

Broadly speaking, the patient was placed in lateral decubitus/ foetal position. If the child was too young or uncooperative, appropriate anaesthesia would be administered for the procedure. For very young children or those with difficult spinal anatomy, the lumbar procedure was performed by an interventional radiologist using imaging guidance. Under asepsis, a spinal needle would be usually introduced at the level of the L3/L4, or L4/L5 interspinous space. Up to 40 drops of CSF (this roughly equated to an estimated 2 ml) and thereafter, collected for required investigations once smooth flow was established. Post-procedure, the patient was kept supine for about 6 hours. During this period, there would be close monitoring of vital signs and the symptoms of post-LP headaches.

### CSF investigation: Cytology method

**Fig 2:**
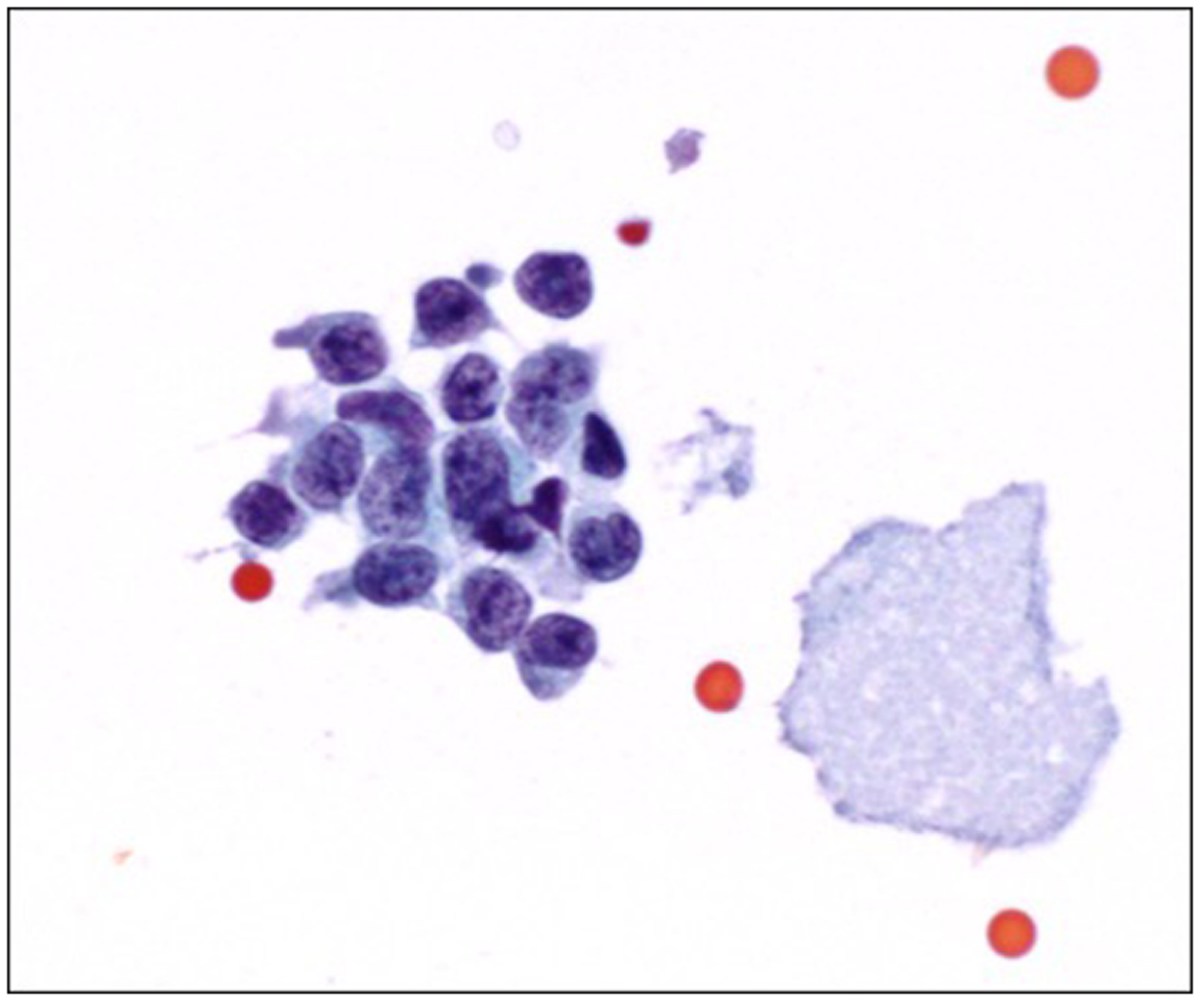
Photomicrograph of cerebrospinal fluid cytology (Papanicolau stain, magnification x1000) shows a cluster of tumour cells that have hyperchromatic nuclei with irregular nuclear contours and coarse chromatin, and scant cytoplasm.

For cytological examination, samples of CSF were mixed with up to 2 drops of BD Surepath^™^ preservative fluid (BD system, USA), placed in a cytospin sample chamber and centrifuged at 600 rpm for 6 minutes. Alcohol-fixed smears (95% ethanol for at least 60 minutes) were placed in an autostainer machine for Papanicolau staining, and air-dried smears were stained using the Hemacolour^®^ stain. Both cytospin samples and stained smears were then reviewed by our in-house pathologists. (Fig 2)

### Statistical analysis

To assess the test performance of EVD or neuroendoscopic (NED) CSF sampling for presence of tumour cells versus the current standard of practice of CSF sampling via LP, the diagnostic odds ratio, sensitivity and specificity of the test were presented. Level of significance was calculated using either chi-square test or Fisher’s Exact test. The sensitivity, specificity, positive predictive value (ppv) and negative predictive value (npv) of CSF cytology from EVD or NED, in comparison to LP, were evaluated against MRI findings of LMD. Statistical significance was set at *p* < 0.05.

## Results

A total of 30 patients were recruited for this study. This cohort consisted of 21 medulloblastoma, 2 intracranial germinoma, 1 pineoblastoma, 1 embryonal tumour with multi-layered rosettes (ETMR), 4 atypical teratoid rhabdoid tumour (ATRT) and 1 ependymoma. Patient ages ranged from 8 months to 15 years old (median age 7 years 2 months), and there were 20 males and 10 females. Intra-operatively, 5 patients had CSF collected via NED and 25 patients had individual EVDs inserted at the start of the surgery. These patients would be henceforth, be referred to as the ‘EVD/ NED’ group. Interval lumbar punctures were performed up to 30 days post-operatively (median time 17.7 days). (Table 1). Based on CSF cytology, 5 patients had corroborative positive CSF cytology on both EVD/ NED and LP procedures. Two patients showed positive cytology only on EVD and 2 had positive cytology from LP. Twenty-one patients demonstrated negative results on both EVD/ NED and LP tests. (Table 2)

**Table 1:** Table depicting patient clinical and tumour demographics, in conjunction with MRI findings, and CSF cytology results from surgery and LP procedures. IG: intracranial germinoma; ATRT: atypical teratoid rhabdoid tumour; ETMR: embryonal tumour with multilayered rosettes.

Patient Number	Age	Sex (Male or Female)	Tumour Histology	Evidence of LMD on MRI: (Yes or No)	Intraoperative (EVD/ NED): CSF cytology postive (Yes or No)	Post-operative LP: CSF cytology postive (Yes or No)	Post-operative LP: Days after surgery
1	2 years 7 months	Male	Medulloblastoma	No	No	No	28
2	8 years 4 months	Male	Medulloblastoma	No	No	No	11
3	12 years	Female	Medulloblastoma	No	No	No	15
4	6 years	Male	Medulloblastoma	No	No	No	18
5	15 years	Male	Medulloblastoma	No	No	No	28
6	14 years	Female	Medulloblastoma	No	No	No	24
7	4 years 3 months	Female	Medulloblastoma	**Yes**	No	**Yes**	17
8	10 years	Male	Medulloblastoma	No	**Yes**	No	21
9	13 years	Female	Medulloblastoma	No	No	**Yes**	13
10	14 years	Male	Medulloblastoma	No	No	No	28
11	7 years	Male	Medulloblastoma	No	No	No	15
12	12 years	Female	Medulloblastoma	No	No	No	14
13	1 year 7 months	Male	Medulloblastoma	No	No	No	14
14	9 years 2 months	Female	Medulloblastoma	No	No	No	10
15	11 years	Male	Medulloblastoma	No	No	No	14
16	6 years 9 months	Female	Medulloblastoma	**Yes**	**Yes**	**Yes**	14
17	5 years	Male	Medulloblastoma	**Yes**	**Yes**	No	14
18	11 years	Female	Medulloblastoma	No	No	No	18
19	1 years 9 months	Male	Medulloblastoma	**Yes**	**Yes**	**Yes**	16
20	13 years	Male	Medulloblastoma	No	No	No	10
21	1 year	Male	Medulloblastoma	No	No	No	10
22	15 years	Male	IG	No	No	No	21
23	12 years	Male	IG	**Yes**	No	No	14
24	2 years	Male	Pineoblastoma	**Yes**	**Yes**	**Yes**	21
25	1 year 10 months	Male	ATRT	No	No	No	20
26	8 months	Male	ATRT	**Yes**	No	No	30
27	1 year 10 months	Female	ATRT	No	**Yes**	**Yes**	21
28	8 months	Male	ATRT	**Yes**	**Yes**	**Yes**	15
29	10 months	Male	Ependymoma	**Yes**	No	No	21
30	1 year 3 months	Female	ETMR	No	No	No	15

**Table 2:** Table showing correlation of cytologic results from 30 pairs of CSF samples from surgery (EVD/ NED) versus LP.

CSF CYTOLOGY	LP		
**EVD/ NED**	Positive	Negative	**Total**
Positive	5	2	7
Negative	2	21	23
**Total number of patients**	7	23	**30**

Following that, 10 patients had MRI findings positive for LMD at the time of diagnosis. In this group, 4 patients had corroborative positive CSF cytology on both EVD/ NED and LP procedures. One patients with positive MRI LMD results had positive cytology from EVD/ NED sampling, but was negative on LP. A total of 3 patients were negative on cytology for both EVD/ NED and LP, despite their MRI scans depicting LMD. (Table 3). Following that, 20 patients did not show evidence of LMD in our study. Seventeen patients in this sub-cohort had corresponding negative CSF cytology findings on both EVD/ NED and LP. However, 1 patient demonstrated CSF cytology positivity for both EVD/ NED and LP, but did not have radiological evidence of LMD in MRI. (Table 4).

**Table 3:** Table showing correlation of MRI results positive for LMD versus corresponding cytology results from CSF samples from surgery (EVD/ NED) and LP, in the same cohort of patients.

MRI positve for LMD	LP		
**EVD/ NED Cytology**	Positive	Negative	**Total**
Positive	4	1	5
Negative	2	3	5
**Total number of patients**	6	4	**10**

**Table 4:** Table showing correlation of MRI results negative for LMD versus corresponding cytology results from CSF samples from surgery (EVD/ NED) and LP, in the same cohort of patients.

MRI negative for LMD	LP		
**EVD/ NED Cytology**	Positive	Negative	**Total**
Positive	1	1	2
Negative	1	17	18
**Total number of patients**	2	18	**20**

Our data analysis shows a statistically significant and strong association between EVD/ NED sampling and LP sampling with a diagnostic odds ratio (OR) of 26.25 (2.94 to 234) (*p* = 0.0034). The CSF sampling via EVD/ NED as a test for evidence of LMD demonstrates a sensitivity of 71.43% and specificity of 91.3%. Based on this, the results imply if EVD/NED sampling is negative, this will be more likely that the LP sampling will be *negative* for tumour cytology. (Table 5). For the correlation between evidence of LMD on MRI findings versus CSF positivity on EVD/ NED or LP, both CSF sampling methods demonstrate significant correlation with MRI diagnostics. The EVD/ NED cohort shows ppv of 71.4% and npv of 78.4%, while the LP group has ppv of 75.0% and npv of 81.8%. Upon subgroup analysis, the LP-based CSF cytology report a higher *p*-value having higher deviation from null hypothesis. (Tables 6 and 7).

**Table 5:** Statistical analysis of CSF sampling from EVD/ NED versus LP.

CSF cytology sampling technique	LP positive cytology	LP negative cytology	OR (95% CI)	*p*-value
**EVD/ NED positive cytology**	5 (71.4%)	2 (8.7%)	26.25 (2.94–234)	0.0034
**EVD/ NED negative cytology**	2 (28.6%)	21 (91.3%)		

**Table 6:** Statistical analysis of MRI results positive for LMD versus corresponding cytology results from CSF samples from surgery (EVD/ NED) and LP, in the same cohort of patients.

MRI findings versus EVD/ NED cytology	MRI positive LMD	MRI negative LMD	OR (95% CI)	*p*-value
**EVD/ NED positive cytology**	5 (71.5%)	2 (28.5%)	9 (1.33–61.14)	0.0164
**EVD/ NED negative cytology**	5 (21.7%)	18 (78.3%)		

ppv = 71.4% and npv = 78.4%

**Table 7:** Statistical analysis of MRI results negative for LMD versus corresponding cytology results from CSF samples from surgery (EVD/ NED) and LP, in the same cohort of patients.

MRI findings versus LP cytology	MRI positive LMD	MRI negative LMD	OR (95% CI)	*p*-value
**LP positive cytology**	6 (75%)	2 (25%)	13.5 (1.95–93.25)	0.0041
**LP negative cytology**	4 (18.2%)	18 (81.8%)		

ppv = 75.0% and npv = 81.8%

## Discussion

Leptomeninges consist of the arachnoid and pia mater and form the boundaries of the subarachnoid space, separated from each other by fine trabeculae and CSF [13]. Tumour cells may gain access and thrive in the subarachnoid space, even without radiological evidence of LMD [3]. Under such circumstances, reliance on ancillary tests for diagnosis is necessary. In 1969, Chang *et al* published a seminal article on the classification of the clinical stages of medulloblastoma, which included tumour dissemination stages (known as the M stages) [14, 15]. Owing to its relevance for patient prognosis, Chang’s M staging has been applied widely for various brain tumours in the field of neuro-oncology [15]. Therefore, the role of CSF sampling to look for tumour cells still maintains its clinical relevance. Previous studies suggest that cytologic examination of lumbar CSF is superior to cytologic examination of CSF obtained from cranial-based shunts (or reservoirs) for detecting LMD in paediatric patients with primary central nervous system (CNS) tumours [1].

The essential goal of CSF diagnostics is to detect neoplastic presence in the CSF for clinicians to react with the most compatible therapy [16]. Early intervention is said to improve quality of life and increase survival to more than 3 months [17]. Current tools available for detecting LMD include CSF cytology and neuroimaging. The latter provides strong support in the diagnosis of tumour spread in patients who have negative results on CSF cytology [18]. Gadolinium-enhanced multi-planar MRI is the imaging modality of choice in suspected cases of LMD [19]. Although there are no definite numbers on the risk of LMD based on tumour type, historical series have demonstrated that medulloblastoma, ETMR and pineoblastoma (previously known as the entity ‘primitive neuroectodermal tumour’ or ‘PNET’) made up the majority of these cases [6, 20]. Atypical teratoid rhabdoid tumour (ATRT)—a rare and hence, lesser understood malignant paediatric brain tumour, has also notoriety for LMD [10, 21]. Others such as ependymoma and germ cell tumours are also associated with LMD [20]. Overall, the risk of LMD is estimated to be between 20% to 50% for these tumour types [20].

Diagnostic gold standard for LMD is cytologic examination of the CSF, although this has limited sensitivity, and currently MRI is frequently the initial study for diagnosing LMD [17, 22]. Leptomeningeal metastasis on MRI classically manifests as abnormal enhancement in the subarachnoid space or along the pia mater [23]. Cranial MRI readings consistent with LMD include ependymal, dural, and leptomeningeal enhancement with, or without resulting hydrocephalus. The spinal MRI screen may reveal linear or nodular enhancement of the cord and thickening of lumbosacral roots [13]. Despite advances in technology, it has been estimated that up to 25% of symptomatic LMD patient can have negative evaluations on MRI imaging [24]. At present, until the time comes for MRI sequences to have enough efficacy to demonstrate microscopic disease, CSF cytology maintains its role for LMD detection. In meantime, continued efforts in bridging radiological and pathological diagnostic gaps needs to be emphasized, especially for paediatric patients with malignant brain tumours. However, until the time comes for current practice to be changed, our study shows that MRI screening maintains a symbiotic role for detecting LMD disease in this cohort of patient.

Currently, CSF cytology is still considered the gold standard for diagnosis of LMD [25]. While it is highly specific (> 95%), it suffers from a lack of sensitivity (< 50%) [25]. Theoretically speaking, we understand CSF circulation to be a dynamic phenomenon—it circulates from the sites of secretion to the sites of absorption according to a unidirectional rostro-caudal flow in ventricular cavities and a multidirectional flow in subarachnoid spaces [26]. Obstructive hydrocephalus secondary to brain tumours is thought to favour ependymal implantation, whereby neoplastic cells becoming attached to the ependymal or leptomeninges at distant sites [1]. These studies have published their efforts to answer the perennial question: whether if CSF sampling from a cranial site versus from a lumbar puncture will show better efficacy for the presence of tumour cytology [1, 19]. In addition, it has been mentioned that to ensure a sufficient amount for accurate analysis, each CSF collection should draw up to 10.5 mL [27, 28]. However, bearing in mind that most of such studies are adult-based, extrapolating such high volumes of CSF may not be feasible in very young, paediatric patients, especially via a LP. In contrast, the use of an EVD/ NED approach will be more likely to produce an ample quantity of starting CSF material for interrogation. In addition, at the *start* of surgery, the previously undisturbed *in situ* tumour mass will be at its maximum volume. Hence, the yield for circulating daughter cells released by the primary tumour will potentially be higher. Next, majority of published papers had the cranial CSF tapped from either Ommaya reservoir or ventriculo-peritoneal shunt (in such cases, presumably from the valve reservoir) [1, 5, 29]. The pertinent difference in our study is the sampling time of the cranial CSF during surgery, as part of an *already planned* operation. If successful, will hence, avoid the need for an additional invasive procedure which may require anaesthesia.

### Study critiques and future work

The authors acknowledge that there are limitations that should be highlighted. First and foremost, this is a retrospective study. Next, the patient population is smaller in comparison to larger, prospective series [1] reported in the literature. This is inevitable as data completeness and adherence to a strict criterion for the purposes of this study was required. Interestingly, our results show CSF cytology to be positive via EVD/ NED but negative when LP is performed. Following that, there are 2 cases positive by LP but negative when CSF is obtained intraoperatively. Nonetheless, we have persuasive results to suggest that positive CSF cytological samples may be sufficient to reflect LMD at the point of surgery. Conversely, for intra-operative ventricular samples that are negative for tumour cells, there is still a role to proceed with an interval lumbar puncture. Based our potential findings, the authors are designing a prospective study to look at higher patient numbers to look at using cranial CSF samples at the time surgery as an acceptable investigative tool for LMD.

## Conclusion

This is a study focused on attempting to answer if direct CSF sampling at the time of naive surgery of malignant paediatric tumours will be a feasible option to avoid a post-operative LP in the same patient. The main objective was to firstly, ascertain if our cranial CSF sampling results will be corroborative with post-operative LP CSF cytology in our local cohort; and next, if this proposed method of CSF sampling can be an acceptable surrogate to replace LP for young children under such disease circumstances. Although the authors are aware that this is a retrospective study with a small population, our data concurs with potential to obliterate an additional procedure for the paediatric patient diagnosed with a malignant brain tumour. In meantime, we strongly advocate continued efforts to elucidate mechanistic pathways for better understanding of LMD in individual tumour types.
